# Alpha sensory stimulation modulates theta phase during speech-print associative learning

**DOI:** 10.1038/s41539-024-00263-5

**Published:** 2024-08-09

**Authors:** Zhijun Liao, Xiya Ao, Yulu Sun, Manli Zhang, Xiangzhi Meng

**Affiliations:** 1 School of Psychological and Cognitive Sciences, Beijing Key Laboratory of Behavior and Mental Health, Peking University, Beijing, 100871 People’s Republic of China; 2 PekingU-Poly U Center for Child Development and Learning, Peking University, Beijing, 100871 People’s Republic of China

**Keywords:** Human behaviour, Reading

## Abstract

Applying 10 Hz (*α-*rate) sensory stimulation, not 5 Hz (*θ*-rate), prior to introducing novel speech-print pairs can reset the phase of *θ* oscillations and enhance associative learning. This rapid gain indicates coordinated mechanisms to regulate attentional/cognitive resources (*α* oscillations) and facilitate memory storage (*θ* oscillations) early in learning. The present findings may inform educational practices for children with reading difficulties.

Learning to associate written with verbal information is essential for reading development. Despite a growing consensus that with reading acquisition, existing brain circuits for visual and speech processing are recycled and integrated^1^, the temporal dynamics of how such print-speech associations are gradually established remain elusive. Our brain can automatically capture the temporal structure in external rhythms through oscillatory entrainment, which is suggested to facilitate perceptual representation^2^ and the formation and storage of unified (multisensory) concepts^3^. Specifically, *α* oscillations are engaged in the modulation of temporally binding and/or conceptually integration (cross-modal) information^4^, while *θ* oscillations are found to support associative memory^5^. Meanwhile, evidence has emerged that behavioral performance also exhibits cyclical fluctuations in a neurophysiologically relevant manner^6^ and is subject to phase alignment induced by sensory input^7^, reflecting the role of designated frequency bands of brain oscillations in specific cognitive processes. The current study thus aimed to investigate whether, and how *α* and *θ* oscillations contribute functionally to speech-print associative learning by applying rhythmic sensory stimulation.

After a unimodal familiarization phase ensuring successful recognition ( > 70%) of the learning materials (5 pseudo-phones and 5 Korean characters), we applied isochronous, synchronized multisensory stimulation, i.e., 16 transient task-irrelevant tone-flash entrainment stimuli, at either 5 Hz (*θ*) or 10 Hz (*α*) before the onset of novel speech-print combinations (i.e., target stimuli with a one-to-one correspondence) in a Paired Associative Learning (PAL; 80 trials × 10 blocks at a congruency-to-incongruency ratio of 1:1) task (Fig. 1a). Thirty-six healthy Chinese undergraduates (no exposure to Korean) were randomly assigned to one stimulation condition and were required to judge whether the character matched the speech sound as accurately and quickly as possible. Correct pairs were learned through feedback. Crucially, a time-resolved approach was adopted to measure the frequency-dependent behavioral effects of rhythmic sensory stimulation by presenting the target stimuli uniformly at one of 40 stimulus onset asynchronies (SOAs; 200–980 ms, in steps of 20 ms) after the onset of the last entrainment stimuli in each trial. In addition to the influence on overall learning accuracy (compared to a *Null*-stimulation control group, *n* = 25), we analyzed whether the phases of *θ* and *α* oscillations, induced by sensory entrainment, modulate the association performance of speech and print at the early exposure (Blocks 1–3) and late consolidation (Blocks 8–10) stages.

**Fig. 1:**
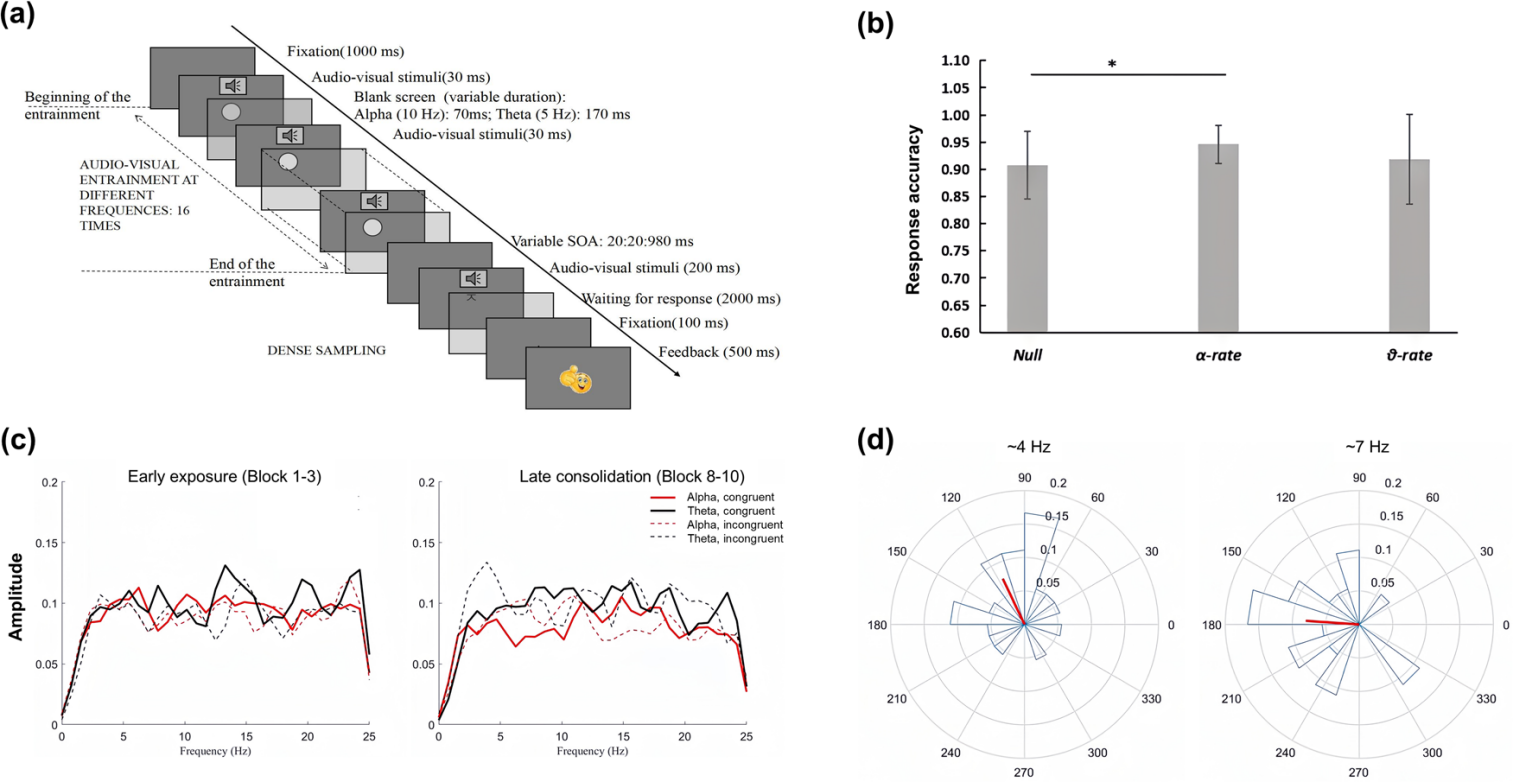
Study protocol and key findings. **a** Experimental design. **b** Response accuracy of paired associative learning for the null, alpha and theta stimulation conditions. Error bars represent ± 1 standard error of the mean. **c** Amplitude spectrum for the detrended reaction time series as a function of frequency during early exposure (Block 1–3; left) and late consolidation (Block 8–10; right) stages. **d** Polar plots for the phase distribution at ~4 Hz (left) and ~7 Hz (right) during the early exposure with alpha-rate stimulation.

First, *α*-rate sensory entrainment (*M* ± *SD* = 0.95 ± 0.03; *t*_*41*_ = −2.39, *p* = 0.021) significantly improved the response accuracy compared to the *Null* condition [0.91 ± 0.06], while *θ*-rate stimulation (0.92 ± 0.08; *t*_*41*_ = 0.60, *p* = 0.595) did not modulate the performance of associative learning (Fig. 1b). To further investigate the influence of external rhythm, normalized reaction time (RT) data were sorted by SOA for the congruent and incongruent trials separately and detrended, then subjected to a fast Fourier transform (zero-padded and multiplexed by a Hanning window). Unexpectedly, neither stimulation induced a significant spectral peak at the corresponding frequency, irrespective of the congruency (Fig. 1c; tested via 5000 times permutation with FDR correction at *q* < 0.05). Based on the result of Rayleigh tests, specifically during the early exposure to congruent speech-print pairs with *α* stimulation, the phases of SOA-aligned RT at ~4 Hz (*r* = 2.015, *z* = 3.345, *p* = 0.034) and ~7 Hz (*r* = 3.075, *z* = 3.469, *p* = 0.030) were not uniformly distributed across participants (Fig. 1d), indicating an initiated phase resetting effects to RT fluctuations. Such an effect was no longer in play at the late consolidation stage, and was neither observed in incongruent trials, nor under *θ*-rate stimulation.

The current finding indicates that *α-* but not *θ*-rate sensory stimulation can reset the phase of *θ* oscillations (4 and 7 Hz) and facilitate the associative learning of speech-print mappings. This aligns with prior research that pre-stimulus *α* oscillations can guide cross-sensory integration^4^, and modulate the memory retention linked with *θ* oscillations^5,8^. Since the phase-resetting we observed was most evident in the initial stage of learning, another putative mechanism is that *α-*rate stimulation may contribute to the suppression of task-irrelevant processing and give rise to theta-band activity that enhances sustained attention to new information^9^. By contrast, the present results provided no evidence that *θ*-rate stimulation would affect speech-print associative learning, which could be due to limited SOA range (compared to one *θ* cycle) and the prolonged interval between two learning stimuli (relative to *α*-rate stimulation). An alternative yet potentially more exciting interpretation is that *θ* oscillations may not be directly involved in the *establishment* of speech-print associations per se, but rather subserve the *maintenance* of associative memory^8^ in the appropriate time frames provided by ongoing *α* oscillations. In other words, the cross-frequency phase-resetting effect observed in our study may suggest *α* and *θ* oscillations as temporally coordinated mechanisms associated with different subprocesses, i.e., the learning (*α*) and memory (*θ*), of speech-print mappings. Given the limited efficiency and signal-to-noise ratio of sensory entrainment, transcranial magnetic/current stimulation combined with online electroencephalography (EEG) recording is recommended in future studies to test our proposed hypothesis and identify the fine-grained temporal dynamics of such interaction. To achieve a more mechanistic understanding, another key question for future research is to investigate how rhythmic (sensory) stimulation may promote learning by modulating attentional processes. Practically, the present observation may lead to learning-based neurophysiological indicators of speech-print mapping difficulty that may benefit the early diagnosis and intervention of developmental dyslexia. In sum, utilizing a novel PAL paradigm with rhythmic sensory stimulation, the present results suggest potentially dissociable functions of *α* and *θ* oscillations in the establishment (i.e., learning) and maintenance (i.e., memory) of cross-modal associations between speech sounds and written symbols.

## Methods

### Participants

Thirty-six right-handed native Chinese speakers (Age range: 18–28 years, *M* = 21.69, *SD* = 2.945; 10 males, 26 females; no Korean language learning experience) from Peking University participated in the current study. All participants had normal or corrected-to-normal vision and hearing levels, without a history of neurological disorder. This study was approved by the Ethics Committee of Peking University. Participants provided written informed consent and received payment (100 RMB) for taking part in the experiment.

### Stimuli

#### Visual stimuli

Counterbalanced across participants, twelve Korean characters (i.e., Hangul; Supplementary Fig. 1) were selected as an approximation of Chinese characters and randomly assigned to the practice (2 characters), cross-modal associative learning task (5 characters), and filler materials in the visual familiarization task (5 characters). Hangul is characterized by its block-based, non-linear composition that greatly differs from other alphabetic writing systems, but bears considerable visuospatial similarity to Chinese radicals. This thus makes Korean characters an ideal tool for investigating speech-print associative learning in Chinese readers.

#### Auditory stimuli

Taking into account the lexical tones, we selected twelve (empty) cells from the “Mandarin Consonants-Vowels Combination Table”^10^ to create pseudo-phones, that is, combinations of consonants, vowels and tones, which can be pronounced as single syllables but do not actually exist in Mandarin Chinese (e.g., “bóu” and “dén”). Speech sounds were synthesized (sampling rate = 44,100 Hz) and normalized in duration (200 ms) and intensity (50 dB) using Praat. The exposure to these pseudo-phones was counterbalanced across participants by randomly assigning them to the practice (2 syllables), cross-modal associative learning task (5 syllables), and filler materials in the auditory familiarization task (5 syllables).

#### Tone-flash entrainment stimuli

Simultaneous presentation of a white solid circle (centered on the screen, size = 3˚, viewing distance = 70 cm, duration = 30 ms) and a synthesized pure tone (sampling rate = 44,100 Hz, frequency = 500 Hz, intensity = 50 dB, duration = 30 ms) constitutes a transient task-irrelevant entrainment stimulus.

The visual and auditory stimuli were presented on a 27” ViewSonic monitor (resolution = 2560 × 1440, refresh rate = 100 Hz) and through headphones, respectively. The familiarization and learning tasks were implemented using Psychtoolbox-3^11^ running on Matlab R2017a (The Mathworks, Inc., Natick, MA, USA).

### Procedure

Experiments started with two unimodal familiarization phases (in random order), followed by the speech-print associative learning task. The overall duration was approximately 50 min.

#### Visual or auditory unimodal familiarization phase

A memory and recognition paradigm was used in the two unimodal familiarization phases to ensure participants’ successful recognition ( > 70%) of the learning materials (5 pseudo-phones and 5 Korean characters). Participants were first instructed to memorize the stimuli (each stimulus was presented 10 times, and the duration was 1 s) with no response required. Then they completed a forced-choice recognition task involving five learned target stimuli and five unlearned fillers by button press, judging whether each item was presented at the memory stage or not (Supplementary Fig. 2). The order of the two unimodal familiarization phases was counterbalanced between subjects.

#### Speech-print associative learning task

A novel time-resolved Paired Associative Learning (PAL) paradigm with rhythmic sensory stimulation was used to probe the role of designated brain oscillations in the acquisition of speech-print associations. Each trial consisted of a sensory entrainment phase (Supplementary Fig. 3, before the red dashed line) and a dense sampling phase (after the red dashed line). Sixteen tone-flash stimuli were presented very briefly (30 ms) and isochronously at a rate of 5 Hz (i.e., the *θ* condition) or 10 Hz (i.e., the *α* condition) in each trial to elicit sensory entrainment before the onset of novel speech-print associations. Participants were randomly assigned to one stimulation condition. To measure the frequency-dependent behavioral effects, a speech-print pair (i.e., the target stimulus) was displayed for 200 ms at a random time interval ranging from 200 to 980 ms in steps of 20 ms after the onset of the last entrainment stimulus. Twenty-five target stimuli were generated by crossing 5 pseudo-phones and 5 Korean characters. In other words, there was a randomly designated one-to-one correspondence for each participant, resulting in 5 congruent and 20 incongruent stimuli. Participants were instructed to judge whether the character matched the speech sound as accurately and quickly as possible during the blank screen (the maximum duration was 2000 ms) by button press (Supplementary Fig. 3). Correct pairs were learned through visual feedback (500 ms). The task consisted of 10 blocks separated by self-terminated breaks for at least 1-minute, involving 80 trials in each block.

An additional learning task *without* prior sensory entrainment was administered as a *Null*-stimulation control task in a separate group of participants (*N* = 25; Age range: 18–26 years, *M* = 21.30, *SD* = 2.483; 10 males, 15 females). Unlike the two rhythmic sensory stimulation conditions, we did not present the tone-flash stimulus repeatedly but only one visual salient stimulus (i.e., the gray solid circle) for 100 ms before presenting the speech-print pair, with a varying SOA (200–980 ms in steps of 20 ms) in between. Moreover, the duration of the first fixation is uniformly selected between 200 and 600 ms in steps of 5 ms. The background color used in this task was white.

### Data analysis

We conducted behavioral data analysis following the procedure in Fiebelkorn et al.^12^ and Song et al.^6^. The overall response accuracy under *α*- or *θ*-rate stimulation was compared against the *Null* condition using an independent samples t-test (two-sided) vis SPSS 20.0 (IBM Corp., 2011). For each participant, trials with RTs > 3 SDs from the subject mean (averaged across congruent and incongruent trials) were excluded from further analysis (see Supplementary Fig. 4 for raw learning curves). We then derived *z*-scores for all remaining RTs to remove interindividual variance in motor responses. RTs after normalization were sorted as a function of ascending SOA for congruent and incongruent trails, respectively (Supplementary Fig. 5). A slowly developing trend typically associated with attention and expectancy was extracted and subtracted from the corresponding RT temporal profiles using the NoiseTools toolbox for Matlab^13^. To convert behavioral time-series data into the frequency domain and measure periodicity in speech-print associative learning, we performed fast Fourier transform (FFT) on the detrended RT data after zero-padding and tapering a Hanning window. To assess statistical significance of the spectral peaks, we further compared them to FFT-transformed surrogate datasets (*N* = 5000) generated by shuffling the detrended RT time series. This resulted in a permutation distribution for each frequency bin, from which we obtained the *p* < 0.05 threshold (FDR corrected for multiple comparison). We also applied circular statistics, i.e., the Rayleigh test^14^, in the CircStats toolbox for Matlab^15^ to examine the nonuniformity of phase values at each frequency bin. Based on visual inspection of error rates and average RTs over individual learning blocks, the first and last three blocks (each consisting of 240 trials) in each experimental condition were selected to represent the early exposure and late consolidation stages of paired associative learning, respectively.

### Reporting summary

Further information on research design is available in the Nature Research Reporting Summary linked to this article.

### Supplementary information

Supplementary Figures

Reporting Summary

## Data Availability

The data that support the findings of this study are available from the corresponding author, Xiangzhi Meng (mengxzh@pku.edu.cn), upon reasonable request.
