# Supplementary material for: Alpha sensory stimulation modulates theta phase during speech-print associative learning
Source: NPJ Sci Learn. 2024 Aug 9;9:51. doi: 10.1038/s41539-024-00263-5 (PMC11315892; doi:10.1038/s41539-024-00263-5)
Supplement: Supplementary file 1 — Supplementary Figures [file 41539_2024_263_MOESM1_ESM.pdf]

Supplementary Figures

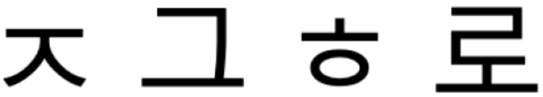

Supplementary Figure 1. Examples of visual materials. Twelve Korean characters (i.e., Hangul; pooled across phonemes and syllables) were selected as an approximation of Chinese characters and randomly assigned to the practice (2 characters), cross-modal associative learning task (5 characters), and filler materials in the visual familiarization task (5 characters).

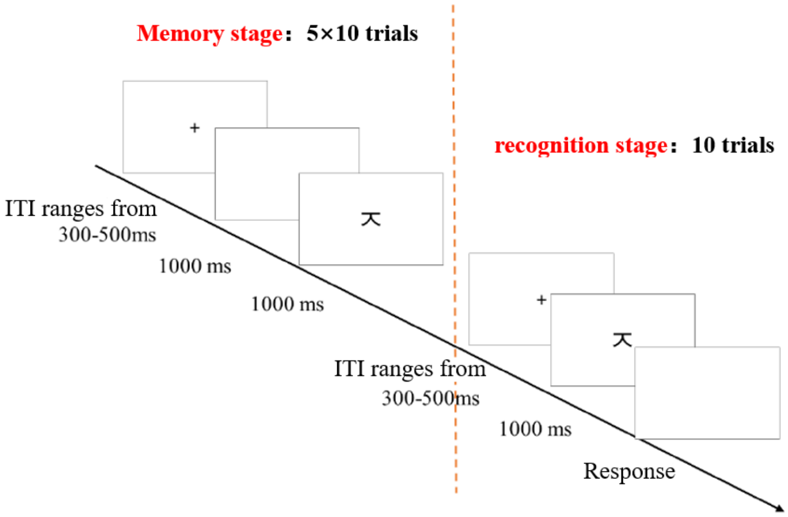

Supplementary Figure 2. Schematic illustration of the visual familiarization phase. The memory stage (before the red dashed line) consists of 50 trials, and the recognition stage (after the red dashed line) consists of 10 trials. ITI = inter-trial interval.

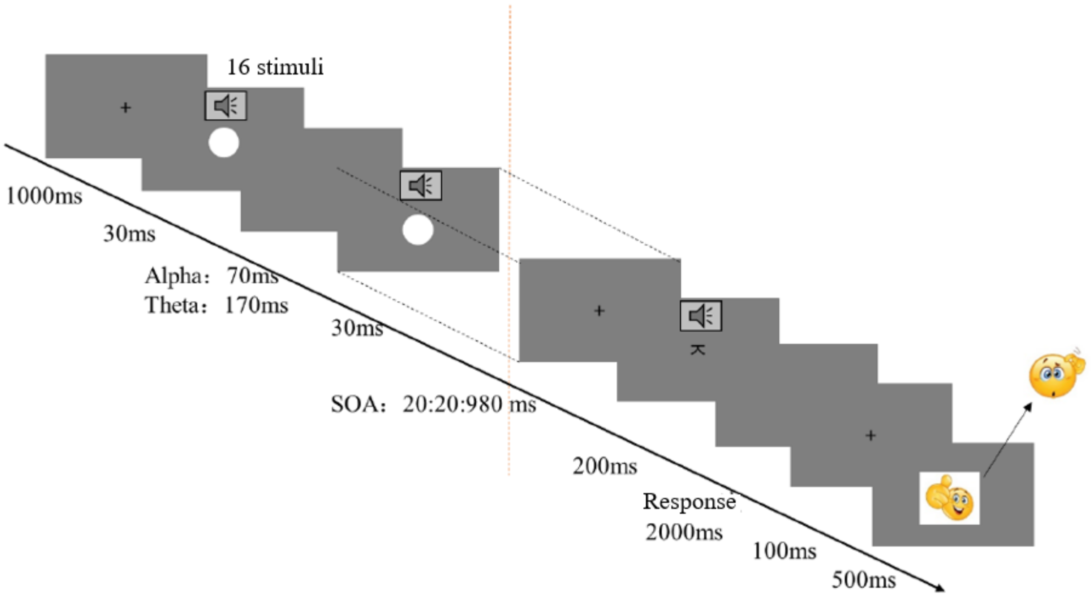

Supplementary Figure 3. Schematic illustration of the time-resolved Paired Associative Learning (PAL) task. Nonlinguistic sensory entrainment (before the red dashed line) was provided prior to the presentation of speech-print pairs with varying stimulus onset asynchronies (SOAs).

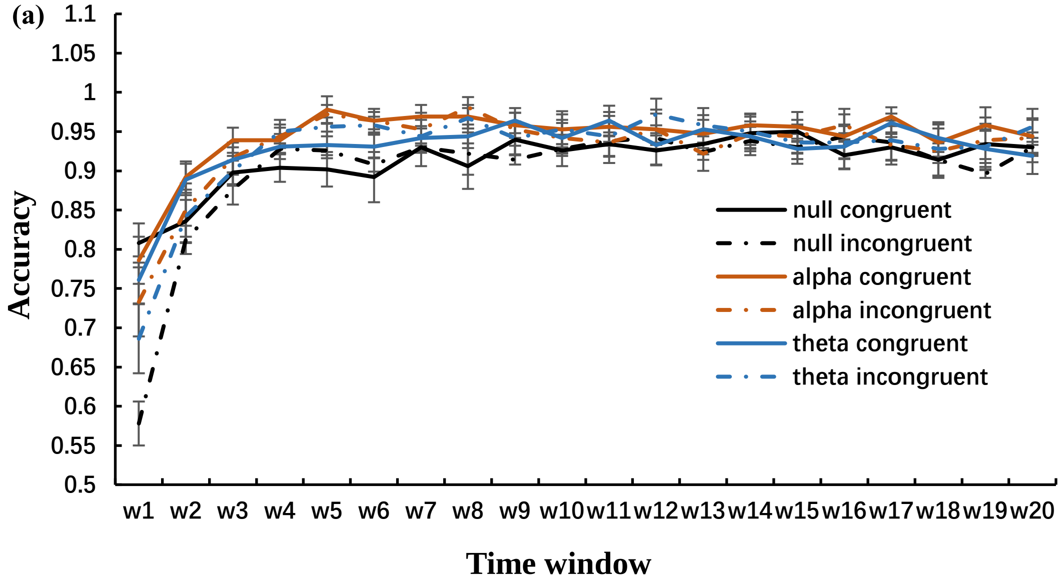

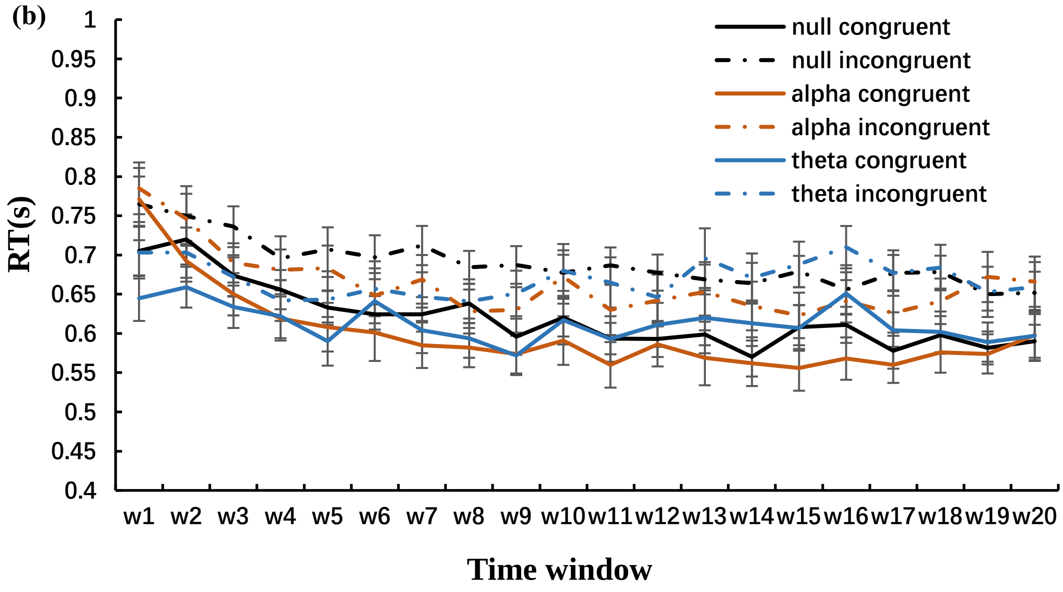

Supplementary Figure 4. The trajectory of speech-print associative learning. It is shown by (a) increasing response accuracy and (b) decreasing reaction times (RT), for the congruent (solid lines) and incongruent (dashed lines) pairs in the null, alpha and theta stimulation conditions. Each time window (wX) consists of forty trials. Error bars represent ± 1 standard error of the mean.

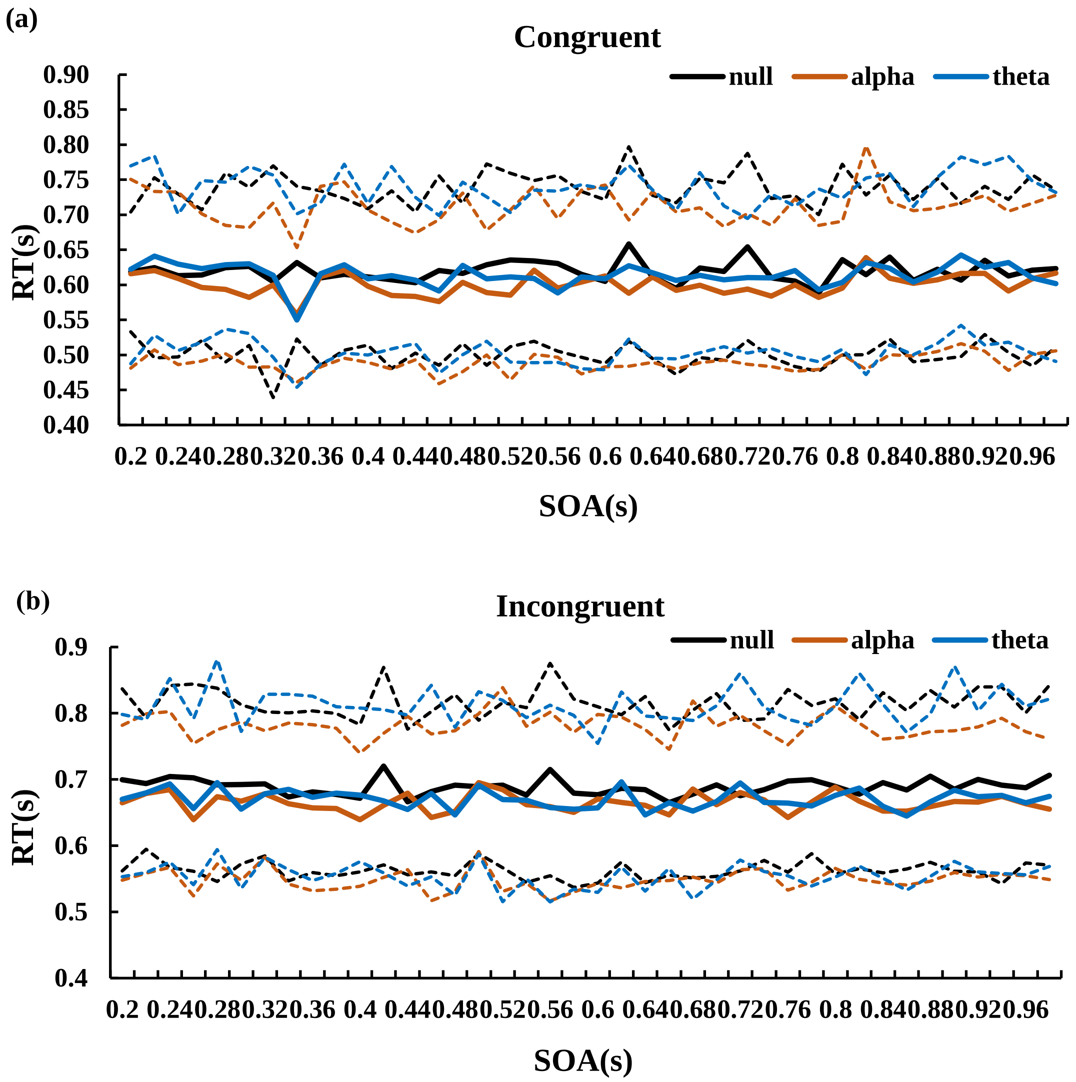

Supplementary Figure 5. Realigned reaction time (RT) time courses as a function of stimulus onset asynchrony (SOA). Solid lines represent the RTs to (a) congruent and (b) incongruent speech-print pairs in the null, alpha, and theta stimulation conditions. Dashed lines indicate ± 1 standard deviation of the mean.
