# Supplementary material for: Alpha sensory stimulation modulates theta phase during speech-print associative learning
Source: NPJ Sci Learn. 2024 Aug 9;9:51. doi: 10.1038/s41539-024-00263-5 (PMC11315892; doi:10.1038/s41539-024-00263-5)
Supplement: Supplementary file 2 — Reporting Summary [file 41539_2024_263_MOESM2_ESM.pdf]

nature portfolio

Corresponding author(s): DAPR, NPJSCILEARN-01060R

Last updated by author(s): Jun 24, 2024

Reporting Summary

Nature Portfolio wishes to improve the reproducibility of the work that we publish. This form provides structure for consistency and transparency in reporting. For further information on Nature Portfolio policies, see our Editorial Policies and the Editorial Policy Checklist.

Statistics

For all statistical analyses, confirm that the following items are present in the figure legend, table legend, main text, or Methods section.

n/a	Confirmed	
☐	☒	The exact sample size (*n*) for each experimental group/condition, given as a discrete number and unit of measurement
☐	☒	A statement on whether measurements were taken from distinct samples or whether the same sample was measured repeatedly
☐	☒	The statistical test(s) used AND whether they are one- or two-sided<br>*Only common tests should be described solely by name; describe more complex techniques in the Methods section.*
☒	☐	A description of all covariates tested
☐	☒	A description of any assumptions or corrections, such as tests of normality and adjustment for multiple comparisons
☐	☒	A full description of the statistical parameters including central tendency (e.g. means) or other basic estimates (e.g. regression coefficient) AND variation (e.g. standard deviation) or associated estimates of uncertainty (e.g. confidence intervals)
☐	☒	For null hypothesis testing, the test statistic (e.g. F, t, r) with confidence intervals, effect sizes, degrees of freedom and P value noted<br>*Give P values as exact values whenever suitable.*
☒	☐	For Bayesian analysis, information on the choice of priors and Markov chain Monte Carlo settings
☒	☐	For hierarchical and complex designs, identification of the appropriate level for tests and full reporting of outcomes
☒	☐	Estimates of effect sizes (e.g. Cohen's *d*, Pearson's *r*), indicating how they were calculated

Our web collection on statistics for biologists contains articles on many of the points above.

Software and code

Policy information about availability of computer code

Data collection	Psychtoolbox-3 running on Matlab R2017a
Data analysis	Matlab function Fast Fourier transform (fft), NoiseTools toolbox for Matlab, CircStats toolbox for Matlab, SPSS 20.0

For manuscripts utilizing custom algorithms or software that are central to the research but not yet described in published literature, software must be made available to editors and reviewers. We strongly encourage code deposition in a community repository (e.g. GitHub). See the Nature Portfolio guidelines for submitting code & software for further information.

Data

Policy information about availability of data

All manuscripts must include a data availability statement. This statement should provide the following information, where applicable:

- Accession codes, unique identifiers, or web links for publicly available datasets
- A description of any restrictions on data availability
- For clinical datasets or third party data, please ensure that the statement adheres to our policy

The data that support the findings of this study are available from the corresponding author upon reasonable request.

Research involving human participants, their data, or biological material

Policy information about studies with human participants or human data. See also policy information about sex, gender (identity/presentation), and sexual orientation and race, ethnicity and racism.

Reporting on sex and gender	Sex and/or gender information was collected based on self-reporting, and consent has been obtained for sharing of individual data. Overall, 20 males and 41 females participated in the current study. No sex/gender-based analysis was performed. Due to the preliminary nature of our study, it might be reasonable to focus on the main effects of sensory stimulation on speech-print associative learning before exploring any additional factors, such as sex and gender. Meanwhile, with a rather small sample size, any sex/gender-based analysis might not yield valid results.
Reporting on race, ethnicity, or other socially relevant groupings	Participants are native Chinese (under)graduate students without further categorization into socially constructed groups.
Population characteristics	Sensory entrainment group (N = 36): 18-28 years, M = 21.69, SD = 2.945. Null stimulation group (N = 25): 18-26 years, M =21.30, SD = 2.483. All participants had normal or corrected-to-normal vision and hearing levels, without a history of neurological disorder.
Recruitment	Participants were recruited from Peking University via social media.
Ethics oversight	The Ethics Committee of Peking University

Note that full information on the approval of the study protocol must also be provided in the manuscript.

Field-specific reporting

Please select the one below that is the best fit for your research. If you are not sure, read the appropriate sections before making your selection.

☐ Life sciences ☒ Behavioural & social sciences ☐ Ecological, evolutionary & environmental sciences

For a reference copy of the document with all sections, see nature.com/documents/nr-reporting-summary-flat.pdf

Behavioural & social sciences study design

All studies must disclose on these points even when the disclosure is negative.

Study description	Quantitative experimental study
Research sample	Peking University (under)graduate students (age range: 18-28 years; 20 males and 41 females), representing typically developing adults.
Sampling strategy	Participants were recruited using a random sampling method from a list of registered students in the university social media. The sample size was determined using a power analysis to ensure sufficient power to detect a medium effect size with an alpha level of 0.05 and a power of 0.80. The chosen sample size of 61 participants is sufficient to achieve reliable and generalizable results, as similar studies in the literature have shown consistent findings with similar sample sizes.
Data collection	The data collection procedure involved the use of a computer. Visual stimuli were presented on a 27” ViewSonic monitor (resolution = 2560 × 1440, refresh rate = 100 Hz), and auditory stimuli were delivered through headphones. The familiarization and learning tasks were implemented and recorded using Psychtoolbox-3 running on Matlab R2017a. During the data collection sessions, only the participant and the researcher were present. The researcher was blind to the experimental conditions and the study hypothesis to minimize bias.
Timing	Data collection was performed between 2020 and 2021.
Data exclusions	Due to technical issues, data collection from two participants was incomplete, and their experimental data were excluded.
Non-participation	No participants dropped out / declined participation.
Randomization	Participants were randomly assigned to experimental conditions by experimenters, taking into account control variables such as age and gender to ensure balanced representation across different experimental conditions.

Reporting for specific materials, systems and methods

We require information from authors about some types of materials, experimental systems and methods used in many studies. Here, indicate whether each material, system or method listed is relevant to your study. If you are not sure if a list item applies to your research, read the appropriate section before selecting a response.

Materials & experimental systems

n/a	Involved in the study
☒	☐ Antibodies
☒	☐ Eukaryotic cell lines
☒	☐ Palaeontology and archaeology
☒	☐ Animals and other organisms
☒	☐ Clinical data
☒	☐ Dual use research of concern
☒	☐ Plants

Methods

n/a	Involved in the study
☒	☐ ChIP-seq
☒	☐ Flow cytometry
☒	☐ MRI-based neuroimaging

Plants

Seed stocks	n.a.
Novel plant genotypes	n.a.
Authentication	n.a.
